# Hypercalcemia related to cholangiocellular carcinoma

**DOI:** 10.1002/ccr3.2286

**Published:** 2019-07-01

**Authors:** Erhan Onalan

**Affiliations:** ^1^ Department of Internal Medicine, Faculty of Medicine Firat Univeristy Elazig Turkey

**Keywords:** cholangiocellular cancer, delta hepatitis, hypercalcemia, malignancie, paraneoplastic

## Abstract

Patients with malignancies may develop hypercalcemia due to bone metastases or paraneoplastic reasons. Hepatocellular cancer should be considered in liver masses detected in chronic viral hepatitis B patients with delta agents. However, in our case, we detected cholangiocellular carcinoma in the etiology of hypercalcemia.

## 1 INTRODUCTION

Patients with malignancies may develop hypercalcemia due to bone metastases or paraneoplastic reasons. Cholangiocellular carcinoma is a rare type of cancer with an incidence of 1‐2/100000. This malignancy may develop anywhere along the biliary tract. A peripheral localization in the liver is unusual for cholangiocellular cancer. Humoral hypercalcemia is uncommon in this malignancy, which is difficult to differentiate from hepatocellular cancer in cases with delta hepatitis. This case presentation reports a case that presented with hypercalcemia and was diagnosed with chronic viral hepatitis B with delta agent and cholangiocellular cancer. Hypercalcemia, which is detected in chronic viral hepatitis B patients with delta agent, and the vast majority of liver masses are associated with hepatocellular cancer. However, this case shows that a rare cause may be cholangiocellular cancer.

Hypercalcemia is the most common metabolic problem in malignancies and threatens survival in patients with cancer. Malignancies constitute the most prevalent cause of hypercalcemia in hospitalized patients.^1^ Approximately 10%‐20% of cancer patients develop hypercalcemia. 85% of these are related to bone metastases. The cause of hypercalcemia in the remaining 15% is malignant humoral hypercalcemia, which is caused by certain cytokines including parathormone‐related peptide (PTHrP) and is usually seen in squamous cell cancers of the head, neck, esophagus, and the lung.^2^ In this report, we present an 83‐year‐old case who received a diagnosis of chronic viral hepatitis B with delta agent and cholangiocellular cancer while being examined for hypercalcemia.

## 2 CASE PRESENTATION

**Figure 1:**
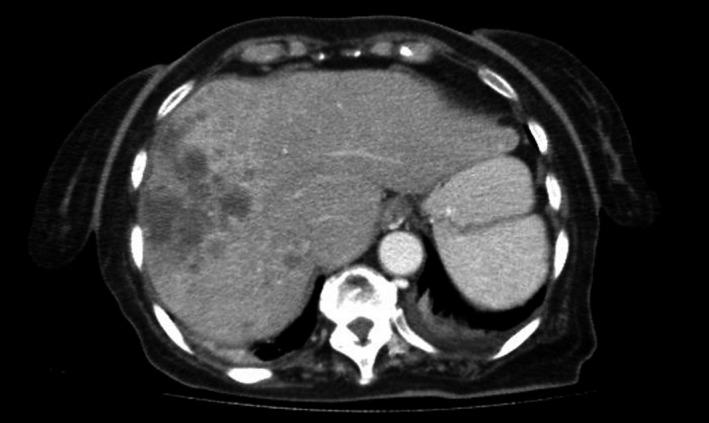
Abdominal tomography demonstrating hypodense lesions with multiple areas of peripheral contrast enhancement congregated particularly in the right lobe of the liver, the largest of which had a size of 47 × 32 mm

An 83‐year‐old female patient presented to our clinic with abdominal pain, lack of appetite, nausea, weight loss, and fatigue. The patient had lost nearly 12 kilograms in the past 6 months. Abdominal pain was localized in the right upper quadrant. She had obtuse pain that had no relation to food. It was found that the patient had osteoporosis in her history but did not receive any treatment. In the physical examination of the patient, who did not present any characteristics in her family history, the liver extended to 2 cm below the costal margin. She did not have ascites or splenomegaly. Heart and lung examinations were normal. Laboratory tests presented a normal hemogram. Biochemical tests revealed an aspartate aminotransferase (AST) level of 50 U/L, alanine aminotransferase (ALT) level of 20 U/L, total bilirubin level of 1.3 mg/dL, alkaline phosphatase (ALP) level of 101 U/L, gamma‐glutamyltransferase level of 227 U/L, albumin level of 2.6 g/dL, total protein level of 4.9 g/dL, calcium level of 12,35 mg/dL, and a phosphate level of 2.6 mg/dL. The patient was hospitalized in order to investigate the etiology of hypercalcemia. The patient had a parathormone level of 9.5 pg/mL. These results excluded a diagnosis of primary hyperparathyroidism. The patient who was thought to have malignant hypercalcemia was started on intravenous fluid replacement and furosemide treatment. The patient who manifested a creatinine clearance of 55.5 mL/min/1.73 m^2^ was started on 2 mg zoledronic acid treatment. Tomography scans demonstrated no osteolytic bone lesions. During follow‐up, the patient's calcium levels regressed to 9.7 mg/dL with the specified treatment. Abdominal ultrasonography performed due to right upper quadrant pain and hepatomegaly revealed multiple hypoechoic images in the liver, of which the largest was 1.5 cm in size, and a coarsely granular parenchyma. The dynamic CT of the patient demonstrated hypodense lesions with multiple areas of peripheral contrast enhancement that were particularly congregated in the right lobe, the largest of which had a size of 47 × 32 mm, and this was interpreted as metastasis (Figure 1).

**Figure 2:**
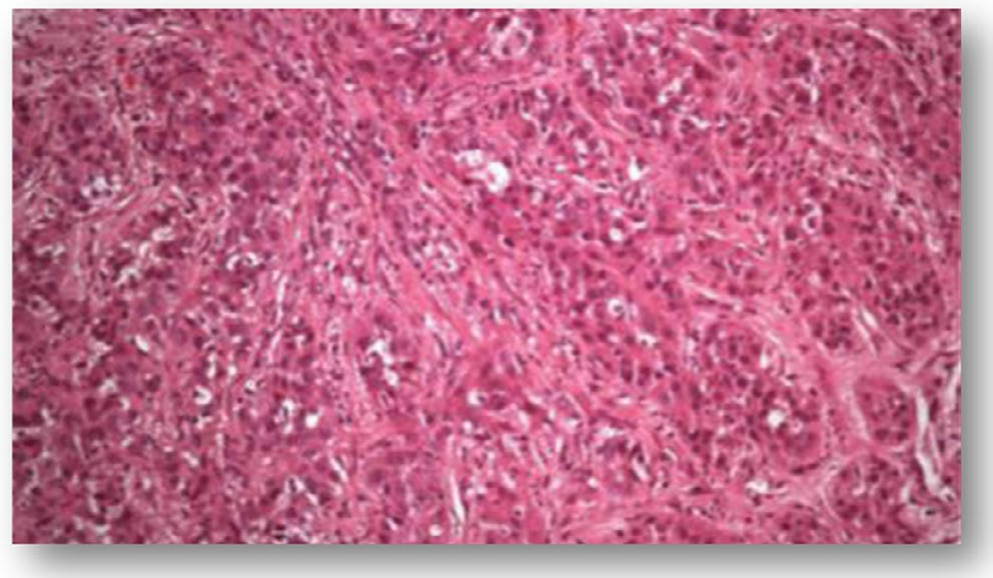
Microscopic appearance of cholangiocellular carcinoma (HE&200)

Levels of alpha‐fetoprotein were determined as 91 ng/mL, cancer antigen (CA‐125) as 258.6 U/mL, carcinoembryonic antigen (CEA) as 0.82 ng/mL, and cancer antigen 19‐9 (CA19‐9) as 2.1 U/mL. A biopsy was performed on the mass in the liver for diagnosis. Pathology result indicated cholangiocellular cancer (Figure 2). The patient did not consent to surgical resection; therefore, a monthly zoledronic acid treatment was planned for pain palpation and hypercalcemia.

## 3 DISCUSSION

Cholangiocarcinoma (CC) is a malignant tumor in the form of adenocancer that originates from the epithelial cells of bile ducts (intrahepatic, hilar, and extrahepatic). Its prevalence is 0.5‐1.2/100000 and it is encountered more frequently in males than in females. The incidence of CC has been increasing gradually, especially in cases of intrahepatic cholangiocarcinoma. The 5‐year survival rate for these patients, including those who received an early diagnosis, is approximately 5%‐10%, and the potential postoperative 5‐year survival rate is 25%‐30%. For cases with metastases, mean survival time does not exceed 8‐12 months, even with medication or combination therapy. The highest incidences of CC are seen in Japan, Chile, East Asia, and India.^3^, ^4^, ^5^ Particularly in cases of CC encountered in western countries, the risk factor is unknown in 90% of the cases, and it is usually attributed to chronic inflammation or biliary irritation. In 10% of the cases, the disease is likely caused by primary sclerosing cholangitis (PSC), obesity, hepatolithiasis, bile stasis‐related cholangitis**,** hepatitis B and C (more in HCV than in HBV), HIV, and parasitic infections (it is endemic in Southeast Asia, and the risk of CC is 14 to 27‐fold higher). Parasitic infestations (particularly Opisthorchis viverrini, Clonorchis sinensis, and Schistosoma japonicum) result in chronic inflammation and increase the risk of CC. Moreover, diabetes mellitus (DM), smoking, advanced age (65% are 65 years old or older), history of biliary surgery, biliary‐enteric anastomosis, chronic inflammatory diseases, being a chronic carrier of typhoid and having cryptosporidiosis, liver cirrhosis (LC), congenital factors (choledochal cysts, Caroli's disease, and congenital hepatic fibrosis), chemical agents (thorotrast, dioxin, nitrosamines and asbestosis), and long‐term use of certain medications (oral contraceptives and isoniazid) may create a risk.^6^, ^7^ The case we have presented did not have any of the strong risk factors defined for cholangiocellular cancer. Our case was diagnosed with chronic viral hepatitis B with delta agent. The mass detected in her liver initially suggested hepatocellular cancer (HCC). She was found to have high alpha‐fetoprotein (AFP) levels, which have an important role in the diagnosis of HCC. CA19‐9 and CA 125 are the most commonly used tests. CA 19‐9 is a weak diagnostic marker with a sensitivity of 40%‐70%, specificity of 50%‐80%, and a positive predictive value (PPV) of 16%‐40%. CA 19‐9 is also higher in PSC and nonmalignant obstructive conditions. CA‐125 levels are high in 65% of these cases.^8^ In a series that involved ninety‐seven patients with cholangiocellular cancer, high CA 19‐9 levels were detected in 28.9% of the patients and CEA levels were high in only 3.1% (13). Our case had normal CA19‐9 and CEA levels. She received a diagnosis of cholangiocellular cancer based on liver biopsy. Hypercalcemia is not rare in patients with cholangiocellular cancer. A study conducted at Mayo Clinic reported hypercalcemia with an unknown cause in 7 of their 40 patients with cholangiocellular cancer.^9^ PTHrP is the most prominent pathophysiological factor‐cause of malignant humoral hypercalcemia.^10^, ^11^ Biochemical symptoms of humoral hypercalcemia include increased serum calcium, low serum phosphate, low PTH, and low 1.25 (OH)2 vitamin D levels.^12^ Our case did not manifest osteolytic bone lesions in her direct radiography, suggesting she did not have bone metastasis. No metastatic bone lesions were detected in her bone scintigraphy. Although metastatic bone involvement cannot be completely eliminated, the fact that it is associated with low phosphate levels suggests that the hypercalcemia seen in this case was probably humoral hypercalcemia of malignancy.

## 4 CONCLUSION

As a result, hepatocellular cancer should be considered in liver masses detected in chronic viral hepatitis B patients with delta agents. However, in our case, we detected cholangiocellular carcinoma in the etiology of hypercalcemia and multiple masses were detected in the liver despite the history of chronic hepatitis B.

## CONFLICT OF INTEREST

None declared.

## AUTHOR CONTRIBUTIONS

EO: conceived the study and wrote, reviewed, and edited the article.
